# A chromosome-level, gap-free genome assembly of the fish parasite *Trypanosoma larimichthysi* from large yellow croaker

**DOI:** 10.1038/s41597-026-07844-6

**Published:** 2026-07-09

**Authors:** Xin Lv, Lanying Liu, Zhideng Zhang, Yang Liu, Wei Huang, Shuigen Li, Yanting Chen, Qinghe Chen, Tuyan Luo

**Affiliations:** 1 Institute of Quality Standards & Testing Technology for Agro-Products, Fujian Academy of Agricultural Sciences, Fuzhou, 350003 China; 2 Fujian Key Laboratory of Agro-products Quality & Safety, Fuzhou, 350003 China; 3 Inspection and Quarantine Technology Center, Fujian Entry Exit Inspection and Quarantine Bureau, Fuzhou, 350003 China; 4 Fujian Provincial Aquatic Technology Extension Station, Fuzhou, 350002 China; 5 School of Breeding and Multiplication, Sanya Institute of Breeding and Multiplication, Hainan University, Sanya, 572025 China

## Abstract

Marine fish trypanosomes are widespread hemoparasites that pose significant threats to wild and farmed teleosts, yet they remain genomically underrepresented compared to their mammalian-infecting counterparts. Here, we present the first chromosome-level, gap-free genome assembly of *Trypanosoma larimichthysi*, a recently described species causing severe trypanosomiasis outbreaks in the economically important large yellow croaker (*Larimichthys crocea*) along the Chinese coast. The assembly, generated using PacBio HiFi long-read sequencing combined with Hi-C chromatin conformation capture, spans 51.04 Mb across exactly 35 pseudochromosomes, with a contig N50 of 1.43 Mb and 97.96% of sequences anchored. Exceptional completeness is evidenced by telomere-to-telomere resolution for 25 chromosomes (58 telomeric loci captured in total), a 99.99% HiFi read mapping rate, and > 99% BUSCO completeness. The genome encodes 10,172 protein-coding genes, with repetitive sequences comprising 50.33%, dominated by retrotransposons including LINE and LTR elements. Comparative genomic analyses confirm the phylogenetic placement of *T. larimichthysi* within *Trypanosoma* and reveal lineage-specific gene family expansions potentially linked to host adaptation and pathogenicity. This reference-grade genome fills a critical gap in aquatic trypanosomatid genomics and provides a valuable resource for investigating parasite evolution, host–parasite interactions, antigenic variation mechanisms, and disease management strategies in mariculture.

## Background & Summary

*Trypanosoma* spp. are flagellated protozoan parasites of the family *Trypanosomatidae* that infect a wide range of vertebrate hosts, including fishes^1–6^. These hemoflagellates are transmitted primarily by leech vectors in aquatic environments and exhibit diverse host specificities, ranging from freshwater to marine species. While mammalian-infecting trypanosomes such as *T. brucei* (causative agent of African sleeping sickness), *T. cruzi* (Chagas disease), and *T. evansi* (surra in livestock) have been intensively studied due to their profound medical and veterinary impacts^7–11^, including mechanisms of antigenic variation, immune evasion, and complex life cycles, fish-infecting trypanosomes remain significantly underinvestigated^12–19^. Fish trypanosomes are prevalent in both freshwater and marine teleosts worldwide, often causing anemia, splenomegaly, tissue damage, and reduced growth, which can lead to substantial mortality in natural populations and intensified losses under aquaculture conditions^12,14,17,20–24^. Despite their ecological and economic relevance, genomic resources for fish trypanosomes are virtually nonexistent, with prior studies limited to morphological descriptions, partial 18S rRNA sequences, and phylogenetic inferences.

The large yellow croaker (*Larimichthys crocea*) is one of the most economically important mariculture species in China, primarily farmed in Fujian and Zhejiang provinces where Ningde accounts for the majority of national production^25–28^. Intensive farming has driven rapid industry growth but also heightened vulnerability to emerging diseases, resulting in significant economic losses^24,29,30^. Since 2023, severe trypanosomiasis outbreaks have affected farmed *L. crocea* along the Chinese coast, with clinical signs including anemia, gill pallor, splenomegaly, and high mortality rates (up to 60% cumulative)^31^. The causative agent has been identified as a lineage closely related to the freshwater parasite *Trypanosoma carassii* (sharing > 98% 18S rDNA homology), initially designated as *T. carassii* spectrum due to its broad host range and cross-salinity adaptation^32^. Recently, it was formally described as a new species, *Trypanosoma larimichthysi* n. sp., based on morphological distinctions (e.g., longer body and centrally located nucleus) and molecular data^31^.

Despite the emerging threat posed by fish-infecting trypanosomes to aquaculture, genomic resources for marine fish trypanosomes remain entirely absent. Currently available Trypanosoma genomes are restricted to mammalian or reptilian parasites (e.g., *T. brucei*, *T. cruzi* and *T. grayi*), and no chromosome-level genome assembly has been reported for any fish-infecting species. High-quality genomic data are essential for resolving phylogenetic relationships, characterizing host adaptation and pathogenic potential, and supporting the development of diagnostic and disease management strategies.

In this study, we report the first near-complete, chromosome-level genome assembly of *T. larimichthysi*, generated using PacBio HiFi long-read sequencing in combination with Hi-C chromatin conformation capture. The assembly spans approximately 51.04 Mb and comprises 50 contigs, with a contig N50 of 1.43 Mb. Using Hi-C data, 97.96% of the assembled sequences were successfully anchored onto 35 chromosomes, resulting in a highly contiguous chromosome-scale assembly. Telomeric repeat analysis further demonstrated extensive assembly completeness, with 25 chromosomes assembled in a telomere-to-telomere (T2T) manner and telomeric repeats detected at 58 chromosomal ends in total. The assembled genome contains 10,172 predicted protein-coding genes, including 469 alternative transcripts. Genome completeness assessment revealed over 99% completeness at both the genome and protein levels, indicating reference-grade assembly quality. Repetitive sequences account for 50.33% of the genome and are dominated by interspersed retrotransposons, particularly LINE and LTR elements, consistent with genomic features observed in other trypanosomatids. In addition, comparative genomic analyses based on single-copy orthologs from representative *Trypanosomatidae* species enabled robust phylogenetic placement and gene family evolution analyses. Together, this dataset represents the first chromosome-level genomic resource for *T. larimichthysi* and provides a high-quality reference for future studies on genome organization, evolution and host–parasite interactions in aquatic trypanosomatids. Importantly, by filling a major genomic gap for fish-infecting trypanosomes, this resource establishes a molecular foundation for genome-informed disease surveillance, risk assessment and long-term health management in large yellow croaker aquaculture.

## Methods

### Sample collection and parasite isolation

**Fig. 1:**
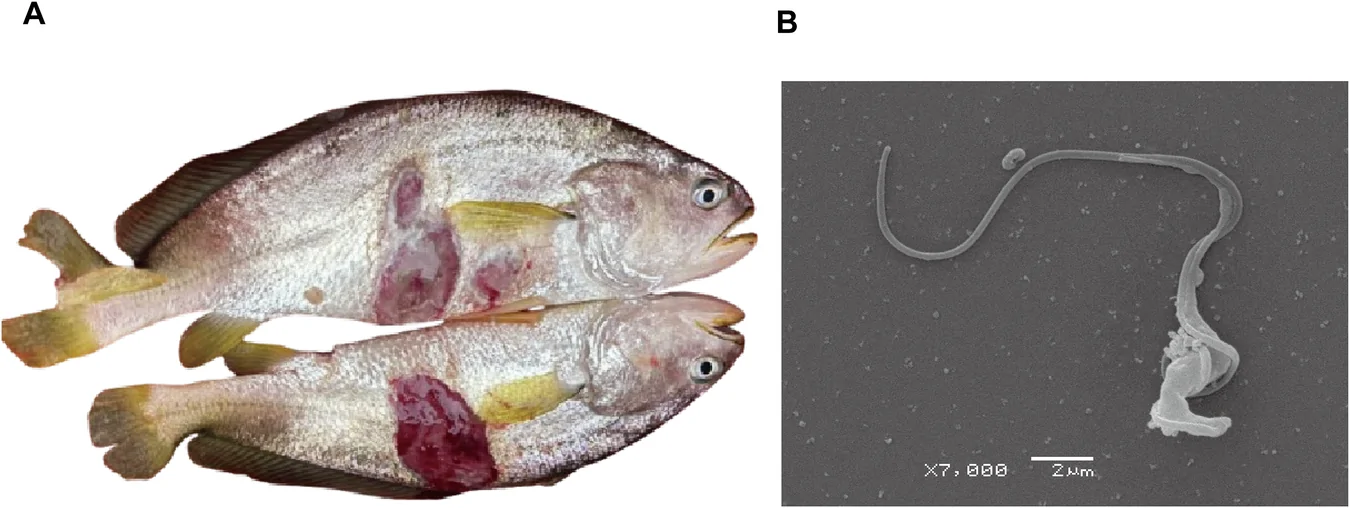
Clinical symptoms of *T. larimichthysi* infection in large yellow croaker and morphology of the parasite. (**A**) Gross pathology of infected *L. crocea*. Two representative large yellow croaker individuals exhibiting typical clinical signs of trypanosomiasis, including extensive hemorrhagic ulcers and necrotic lesions on the body surface. (**B**) Scanning electron micrograph of a purified *T. larimichthysi* trypomastigote. The parasite displays characteristic elongated morphology with a prominent undulating membrane, posterior kinetoplast, and free flagellum. Scale bar: 2 μm. Magnification: × 7,000.

Blood samples were collected from *L. crocea* exhibiting typical symptoms of trypanosomiasis, including lethargy, anemia, and extensive hemorrhagic ulcers with necrotic lesions on the body surface (Fig. 1A). Fish were sourced from a net cage aquaculture facility in the Baimatan area of Sanduao Bay, Jiaocheng District, Ningde City, Fujian Province, China. Blood was drawn from the caudal vein using sterile syringes and immediately transferred into tubes containing EDTA-K₂ as an anticoagulant. Samples were transported to the laboratory on ice.

*T. larimichthysi* parasites were isolated and purified using Percoll density gradient centrifugation. The Percoll separation solution was prepared by mixing Percoll stock solution with PGSS buffer (57 mmol/L Na₂HPO₄, 3 mmol/L NaH₂PO₄, 44 mmol/L NaCl, 4 mmol/L KCl, 5 mmol/L glucose, 80 mmol/L sucrose, pH 7.4) at a volume ratio of 5.1:4.9. Fresh anticoagulated blood was mixed with an equal volume of Percoll separation solution and centrifuged at 800 × g for 15 min at 4 °C. The upper layer above the red blood cell pellet, including the plasma supernatant, the leukocyte-rich interface layer (containing enriched *T. larimichthysi*), and a small amount of red blood cells, was carefully aspirated. This upper fraction was then mixed with an equal volume of fresh Percoll separation solution and centrifuged again at 1000 × g for 10 min at 4 °C. The lower layer, highly enriched in motile *T. larimichthysi* trypomastigotes (Fig. 1B), was collected and used for subsequent *in vitro* culture.

### *In vitro* culture of *T. larimichthysi*. 

The purified *T. larimichthysi* pellet was resuspended in RPMI 1640 complete medium supplemented with 10% (v/v) fetal bovine serum (FBS) and 1% (v/v) penicillin-streptomycin solution (100 U/mL penicillin, 100 μg/mL streptomycin). The suspension was maintained in a cell culture incubator at 26–28 °C with 5% CO₂. Parasite growth and density were monitored daily using an inverted microscope. Medium was replaced periodically as needed to purify and expand the parasite population.

### Scanning electron microscopy

Purified *T. larimichthysi* trypomastigotes from *in vitro* culture were processed for scanning electron microscopy as follows. Samples were fixed in 1.5% glutaraldehyde for 4 hours at room temperature. After three washes in phosphate buffer (10–15 min each), samples were post-fixed in 1% osmium tetroxide for 4 hours. Following three washes in distilled water (10–15 min each), samples were dehydrated through a graded ethanol series (50%, 70%, 80%, 90%, and 100%; 10–15 min each step, with three changes at 100%). Dehydrated samples were then substituted with propylene oxide (two changes), followed by critical-point drying using a Hitachi HCP-2 critical point dryer. Dried specimens were mounted on stubs, sputter-coated with gold using an EIKO IB-5 ion coater, and examined with a JEOL JSM-6380LV scanning electron microscope at an accelerating voltage of 15–20 kV. Representative images were captured (Fig. 1B).

### Total DNA extraction from *T. larimichthysi*

Log-phase pure cultures of *T. larimichthysi* were centrifuged at 1000 × g for 10 min at 4 °C to pellet the parasites. Total genomic DNA was extracted using the Bacterial Genomic DNA Extraction Kit (Tiangen Biotech, Beijing, China) according to the manufacturer’s instructions. DNA concentration and purity were assessed using a NanoDrop spectrophotometer. Integrity was verified by 1% agarose gel electrophoresis. Qualified DNA samples were stored at −20 °C for long-term use or −80 °C for archiving.

### PacBio HiFi library preparation and sequencing

High-molecular-weight genomic DNA extracted from *T. larimichthysi* was used as input for PacBio HiFi library construction. The library was prepared using the SMRTbell® Express Template Prep Kit 2.0 (Pacific Biosciences, Menlo Park, CA, USA) according to the manufacturer’s instructions. Briefly, 5–10 μg of input DNA was sheared to a target fragment size of 15–20 kb using a Megaruptor 3 system (Diagenode, Belgium). Sheared DNA underwent damage repair, end repair, and A-tailing, followed by ligation of SMRTbell adapters and nuclease treatment to remove failed ligation products. Size selection was performed to enrich fragments ≥ 15 kb using AMPure PB beads or the Short Read Eliminator kit. Library quality was assessed using a Femto Pulse system (Agilent Technologies, Santa Clara, CA, USA) to confirm the target size distribution. Final library concentration was quantified by Qubit fluorometry (Thermo Fisher Scientific, Waltham, MA, USA).The library was sequenced on the PacBio Sequel II system using HiFi chemistry with 24–30 hour movie times. Circular consensus sequencing (CCS) reads were generated using SMRT Link software (v11 or later), requiring a minimum of 10–15 passes per molecule and predicted accuracy ≥ Q20. Finally, a total of 4.96 Gb of high-quality HiFi data was generated, comprising over 250,000 reads with most reads ranging from 10,000 to 30,000 bp. The mean read length was 19,778 bp, the longest read was 59,469 bp, the shortest was 372 bp, and the N50 read length was 20,410 bp.

### Hi-C library preparation and sequencing

For chromosome-level scaffolding, a Hi-C library was prepared from the same high-quality *T. larimichthysi* genomic DNA sample to preserve native chromatin conformation. The Hi-C library was constructed using the Arima-HiC + Kit (Arima Genomics, San Diego, CA, USA) following the manufacturer’s protocol. Briefly, DNA was crosslinked with formaldehyde, digested with a restriction enzyme cocktail, and proximity-ligated with biotinylated bridge adapters. Proximity-ligated DNA was sheared to 300–600 bp using a Covaris sonicator, followed by biotin enrichment, end repair, A-tailing, and Illumina-compatible adapter ligation using the KAPA HyperPrep Kit (Roche). The library was amplified with the minimum number of PCR cycles determined by qPCR-based optimization to avoid over-amplification. Final library quality, size distribution, and complexity were evaluated using a TapeStation system (Agilent) and Arima-specific QC assays. The Hi-C library was sequenced on an Illumina NovaSeq 6000 platform in paired-end 2 × 150 bp mode, generating sufficient read pairs (typically > 100 million) for high-resolution contact mapping and chromosome-scale scaffolding.

### RNA-Seq library preparation and sequencing

RNA sequencing was performed to support genome annotation. Total RNA was extracted from mixed *T. larimichthysi* samples using the TRIzol reagent (Invitrogen, USA) following the manufacturer’s instructions. RNA quality and integrity were assessed using agarose gel electrophoresis and a Qubit fluorometer (Thermo Fisher Scientific, USA). A cDNA library was constructed using a standard Illumina RNA library preparation protocol. After library quality assessment, paired-end sequencing was carried out on the Illumina NovaSeq 6000 platform, generating high-quality transcriptomic reads for genome annotation.

### Genome assembly and quality assessment

Primary contig assembly was performed using the PacBio HiFi reads with Hifiasm^33^ (v0.25) run with default parameters. To reduce small remaining gaps in the assembly, TGS-GapCloser^34^ (v1.2.1) was applied using the following parameters: -m 2000 -c 2 -l 100 -s minimap2 -p 80. Chromosome-level scaffolding was achieved by incorporating the Hi-C data. Hi-C reads were processed using HiC-Pro^35^ (v3.1.0) with default parameters, which included quality control, trimming, alignment to the Hifiasm contigs using Bowtie2^36^. Only uniquely mapped read pairs were retained for downstream analysis. Valid interaction pairs were identified through HiC-Pro’s standard filtering pipeline, and genome-wide contact matrices were generated at multiple resolutions. The resulting Hi-C contact maps were used for genome scaffolding and manual curation. Initial chromosome-scale scaffolding was performed based on the Hi-C interaction signals, followed by inspection and correction of misassemblies using Juicebox Assembly Tools^37^ (v2.20.00), ensuring accurate chromosome structure and orientation. he final chromosome-scale assembly was visualized using Circos^38^ (v0.69-9) to display genome-wide Hi-C interaction heatmaps and confirm proper long-range scaffolding. Telomeric regions were identified by searching the assembly for tandem repeats using Tandem Repeats Finder^39^ (TRF, v4.09) with the parameters ‘2 6 6 80 10 50 2000’. Repeats consisting primarily of the motifs TTAGGG or CCCTAA present in long tandem arrays at chromosome ends were annotated as telomeric regions. Genome completeness was assessed using Benchmarking Universal Single-Copy Orthologs (BUSCO, version 5.5.0)^40^ in genome or protein mode with the Euglenozoa_odb10 dataset. To evaluate assembly accuracy, the original HiFi reads were realigned to the final chromosome-scale assembly using Minimap2 (v2.26) with the preset -ax asm20, followed by sorting and indexing with SAMtools (v1.19)^41,42^.

**Fig. 2:**
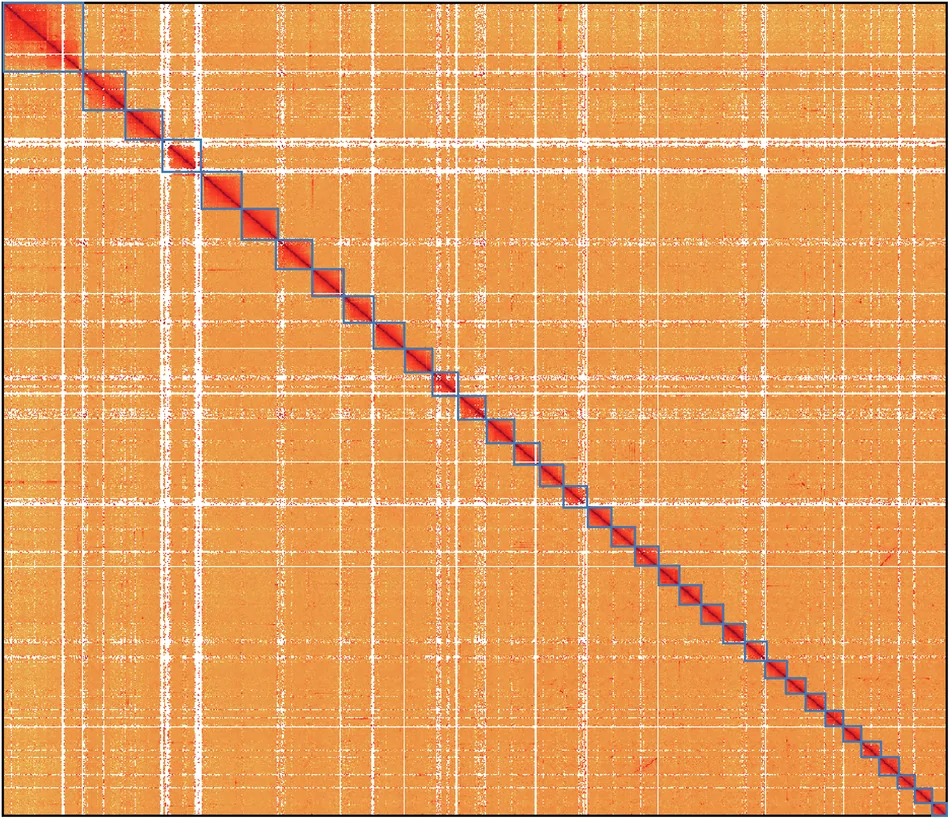
Genome-wide Hi-C interaction heatmap of the *T. larimichthysi* chromosome-level assembly. Hi-C contact matrix showing long-range chromatin interactions across the 35 pseudochromosomes of *T. larimichthysi*. The heatmap was generated using contact data binned at 50 kb resolution. Strong diagonal signals indicate robust intra-chromosomal interactions, with clear boundaries delineating individual chromosomes, confirming successful chromosome-scale scaffolding. Color intensity represents the normalized interaction frequency (orange: low; red: high).

**Fig. 3:**
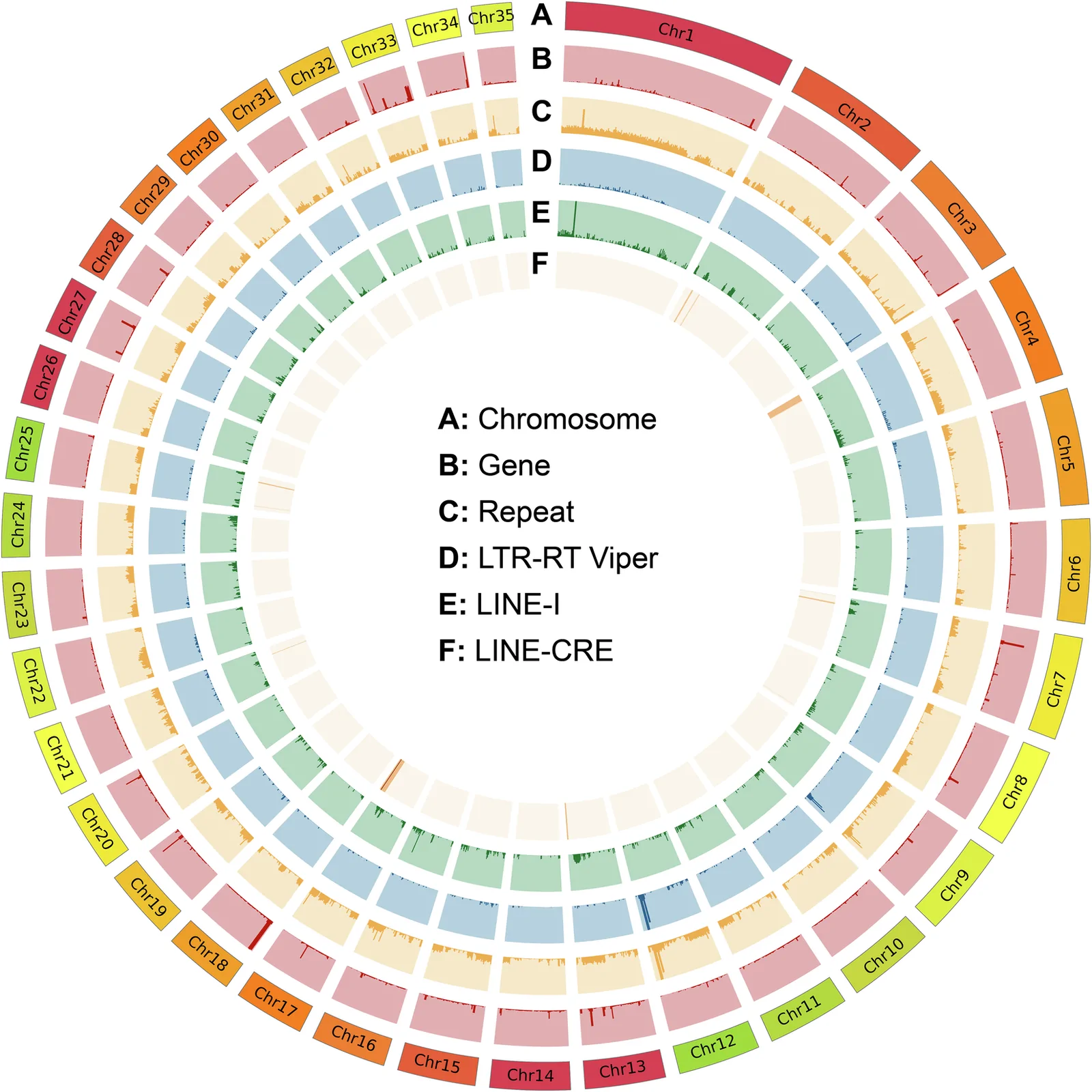
Circular representation of the *T. larimichthysi* chromosome-level genome assembly. The circos plot displays the 35 pseudochromosomes arranged in order of decreasing size (Chr1 largest, Chr35 smallest). Tracks from outer to inner are as follows: (A) Chromosome ideograms (colored by chromosome); (B) Gene densit; (C) Repeat density; (D) Density of LTR-RT Viper elements; (E) Density of LINE-1 elements; (F) Density of LINE-CRE elements. Densities were calculated in 20 kb non-overlapping windows. The plot highlights the distribution of genes and various classes of transposable elements across the genome.

The primary HiFi-based assembly yielded a highly contiguous draft genome comprising only 50 contigs with a total size of approximately 51 Mb, a contig N50 of 1.43 Mb, and a contig N90 of 963 kb (Table 1). Subsequent Hi-C-assisted scaffolding successfully anchored 97.96% of the assembly into 35 pseudochromosomes (Table 1; Figs. 2, 3). The genome-wide Hi-C interaction heatmap exhibited strong, block-like diagonal signals with sharp boundaries and minimal inter-chromosomal interactions, confirming the high accuracy and reliability of the chromosome-scale scaffolding (Fig. 2). Following gap-filling procedure, all remaining gaps were closed, resulting in a final gap-free, chromosome-level genome consisting of 35 pseudochromosomes. The original PacBio HiFi reads were realigned to validate the continuity and accuracy the final assembly. A mapping rate of 99.99% was achieved, indicating near-perfect concordance between the reads and the assembly.

**Table 1:** Genome assembly statistics and annotation summary for *T. larimichthysi*.

Genome Features	Values
Genome size (bp)	51,039,137
Contig Number	50
Contig N50 (bp)	1,432,669
Contig N90 (bp)	963,253
Num of Chromosome	35
Anchoring Rate	97.96%
Repeat Content	50.33%
Gene number	10,172
Alternative Transcript	469
GC	45.24%
BUSCO	99%

To evaluate potential host contamination, all PacBio HiFi reads used for genome assembly were aligned against the telomere-to-telomere genome assembly of the large yellow croaker (*Larimichthys crocea*; accession GWHGDII00000000.1) using minimap2 with default parameters^31^. Only 446 of 250,777 HiFi reads (0.178%) were identified as primary alignments to the host genome, indicating minimal host-derived sequences in the dataset. These results suggest that host contamination was negligible and did not substantially affect the assembly.

Notably, of the 35 chromosomes, 25 achieved complete telomere-to-telomere (T2T) assembly with telomeric repeats detected at both ends, 8 had telomeric repeats at one end, and only two chromosomes (Chr13 and Chr30) lacked detectable telomeric repeats at either end (Table 2). In total, 58 telomeric repeat loci were captured, representing one of the most complete telomeric resolution reported to date for trypanosomatid genome. Additionally, BUSCO analysis revealed over 99% of genome completeness of the *T. larimichthysi* assembly. In summary, the final chromosome-level genome of *T. larimichthysi* represents a highly contiguous and complete genome assembly, providing an important reference resource for trypanosomatid genomics. (Table 1; Figs. 2, 3).

**Table 2:** Telomere annotation for 35 chromosomes of *T. larimichthysi*.

Chr ID	Chr Length	Num of TR in 5’ End	Num of TR in 5’ End	Chr ID	Chr Length	Num of TR in 5’ End	Num of TR in 5’ End
**Chr1**	4,129,302	190	232	**Chr19**	1,242,618	399	59
**Chr2**	2,508,586	301	0	**Chr20**	1,231,398	229	209
**Chr3**	2,113,686	406	314	**Chr21**	1,192,928	0	93
**Chr4**	2,078,885	49	115	**Chr22**	1,185,670	252	160
**Chr5**	2,069,646	323	90	**Chr23**	1,177,662	48	0
**Chr6**	1,857,461	273	167	**Chr24**	1,136,196	293	132
**Chr7**	1,839,580	692	74	**Chr25**	1,100,437	414	139
**Chr8**	1,799,459	133	0	**Chr26**	1,078,860	117	217
**Chr9**	1,614,900	0	139	**Chr27**	1,037,357	133	0
**Chr10**	1,552,580	172	407	**Chr28**	1,002,119	355	226
**Chr11**	1,463,024	460	167	**Chr29**	983,677	94	121
**Chr12**	1,462,281	291	0	**Chr30**	980,522	0	0
**Chr13**	1,432,669	0	0	**Chr31**	965,516	212	175
**Chr14**	1,428,715	150	213	**Chr32**	963,253	268	133
**Chr15**	1,389,899	326	171	**Chr33**	937,282	71	164
**Chr16**	1,336,930	331	0	**Chr34**	918,063	58	49
**Chr17**	1,257,987	171	56	**Chr35**	710,680	246	257
**Chr18**	1,254,402	377	120				

### Gene prediction and functional annotation

Gene structure prediction was performed using GETA (v2.5.7; https://github.com/chenlianfu/GETA), a pipeline that integrates *ab initio* prediction, homology-based searching, and transcriptome-guided assembly. Specifically, GETA incorporates multiple tools including AUGUSTUS^43^ (v3.3.3) for *ab initio* gene prediction, Exonerate^44^ (v2.4.0) and GeneWise^45^ for homology-based alignment of protein sequences from related species, PASA^46^ (v2.4.1) for transcriptome-based evidence alignment using Hisat2^47^ (v2.2.1) and StringTie (v2.2.1)^48^, and HMMER^49^ (v3.3) for domain-based searches. Functional annotation of the predicted proteins was conducted using InterProScan^50^ (v5.60-92.0) to identify protein domains and motifs against the Pfam^51^, Gene Ontology^52^ (GO), and other integrated databases. Orthology and additional functional assignments were obtained using eggNOG-mapper^53^ (v2.1.6) with the eggNOG database (v5.0), enabling mapping to Kyoto Encyclopedia of Genes and Genomes^54^ (KEGG) pathways, Clusters of Orthologous Groups^55^ (COG), and Swiss-Prot^56^ annotations.

The final consensus gene set comprised 10,172 protein-coding genes, among which 469 genes produced alternative transcripts (Table 1). Completeness of the annotation was evaluated using BUSCO, revealing over 99% completeness, confirming the high integrity and accuracy of the predicted gene models. Functional annotation results showed that 7,281 genes (71.6% of the predicted genes) received functional assignments, primarily from the NR, COG, Pfam, KEGG, and GO databases, annotating 5,524, 5,524, 5,129, 2,150, and 458 genes, respectively.

### Annotation of repetitive sequences

De novo identification of repetitive elements was performed using RepeatModeler^57^ (v2.0.3), which generates a consensus library of repeat families through self-comparison and modeling. The resulting library was used as a custom database for RepeatMasker^58^ (v4.1.0) to mask and classify repetitive regions in the *T. larimichthysi* genome assembly.

Repetitive sequences accounted for 50.33% of the *T. larimichthysi* genome (Table 1). Nearly all repeats were interspersed (46.67% of the genome), with retrotransposons dominating the repetitive landscape. The most abundant classes were non-LTR retrotransposons of the LINE/I (Jockey) family, represented by 1,790 copies comprising 12.91% of the genome, and LTR retrotransposons of the Viper family, a trypanosomatid-specific endogenous viral element family, with 892 copies covering 5.50% of the genome (Fig. 3, tracks D–F).

### Phylogenomic Inference and Gene Family Evolution

To determine the evolutionary position of *T. larimichthysi* within the *Trypanosomatidae* family, we conducted a comparative genomic analysis including 19 representative trypanosomatid species and *Bodo saltans* as an outgroup. Orthologous gene groups were inferred using OrthoFinder^59^ (v2.5.4), which combines sequence similarity searches and phylogenetic reconciliation to identify orthologs and paralogs across species. Single-copy orthologous genes were extracted, aligned individually with MAFFT^60^ (v7.526) using the–auto parameter for optimal alignment strateg. The alignments were then concatenated into a supermatrix containing 1,274 single-copy orthologs. Phylogenetic reconstruction was performed under the maximum likelihood framework using RAxML^61^ (v8.2.12), running 100 bootstrap replicates for branch support. The resulting tree was visualized using iTOL^62^ (v5). Divergence times were estimated using MCMCTree from the PAML package (v4.9i)^63^ in a Bayesian framework. The analysis incorporated a relaxed molecular clock with uncorrelated rates, using two soft-bound fossil calibration points from TimeTree^64^. Gene family expansion and contraction were analyzed using CAFE^65^ (v4.2.1) on the OrthoFinder output, with a significance threshold of p < 0.01. This identified significant changes in gene family sizes along the phylogenetic tree, revealing evolutionary adaptations in *T. larimichthysi*. To investigate the functional significance of significantly expanded gene families, KEGG pathway enrichment analysis was performed using Fisher’s exact test. Multiple testing correction was applied using the Benjamini–Hochberg false discovery rate (FDR) method with the p.adjust function in R (v4.3.0). Enriched pathways were visualized as bubble plots using ggplot2^66^ (v3.4.2)

**Fig. 4:**
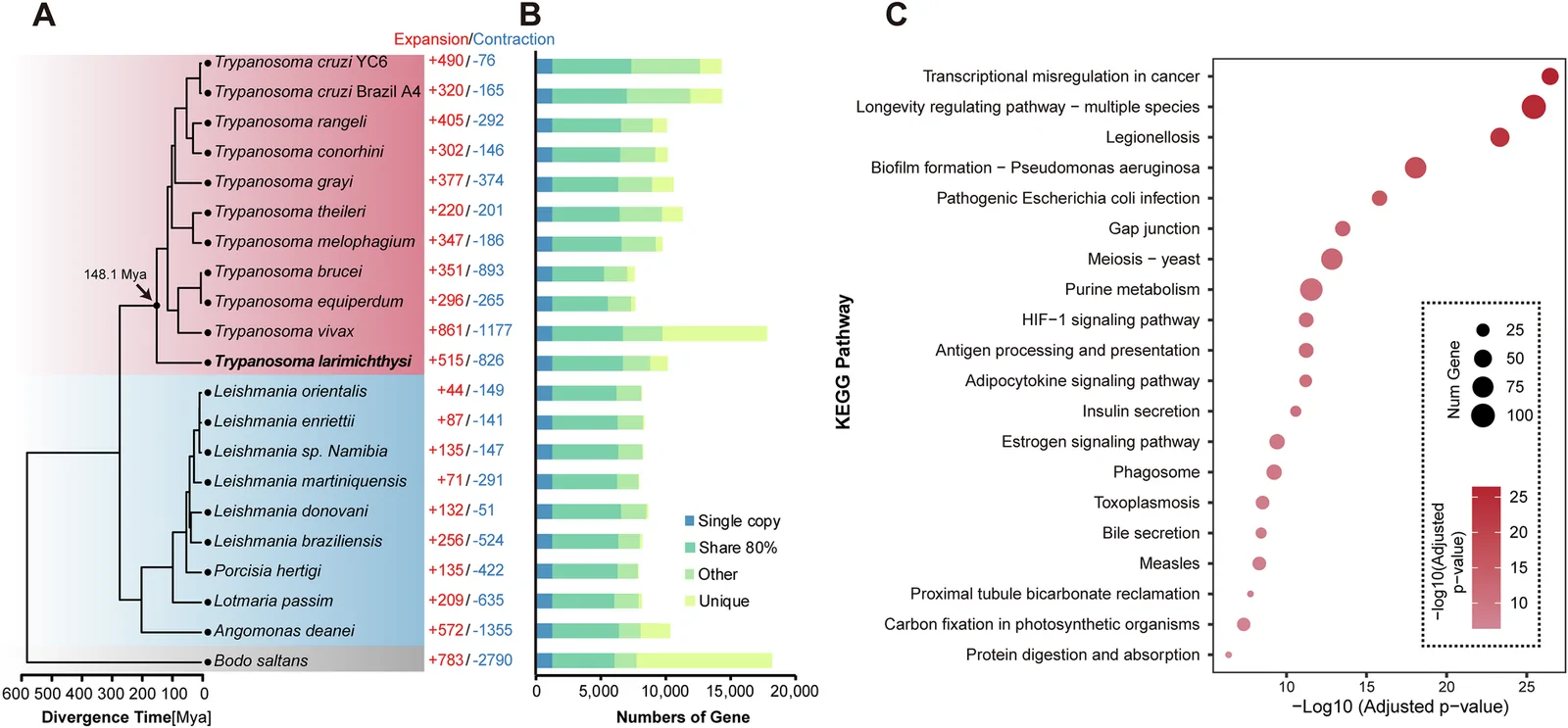
Phylogenetic position, gene family evolution, and functional enrichment of expanded genes in *T. larimichthysi*. (**A**) Maximum likelihood phylogenomic tree of *T. larimichthysi* and 19 related trypanosomatid species, with *B. saltans* as the outgroup. The tree is based on a concatenated alignment of 1,274 single-copy orthologous proteins. The timescale (Mya) at the bottom represents estimated divergence times. (**B**) Gene family expansion and contraction across *Trypanosomatidae* (expansions in red, contractions in blue). Bar charts illustrate the proportion of single-copy, shared ( ≥ 80% species), other, and unique orthologous groups for each species, highlighting overall conservation of gene families among these species. (**C**) KEGG pathway enrichment analysis of significantly expanded gene families in *T. larimichthysi*. Bubble plot showing enriched pathways (Fisher’s exact test, adjusted p < 0.05), ordered by significance. Dot size represents the number of genes in the pathway, and color intensity reflects -log_10_(adjusted p-value).

Phylogenetic reconstruction based on 1,274 single-copy orthologous genes placed *T. larimichthysi* firmly within the genus *Trypanosoma*, forming a monophyletic clade with other fish-infecting and mammalian trypanosomes (Fig. 4A). Divergence time estimation indicated that *T. larimichthysi* diverged from its closest relatives approximately 144–149 million years ago (Mya). Gene family analysis revealed that the majority of orthologous groups are relatively conserved across *Trypanosomatidae* species (Fig. 4B). Gene family expansion and contraction analysis exhibited 515 and 826 gene families that were expanded and contracted in the *T. larimichthysi* genome, respectively. KEGG pathway enrichment analysis of the significantly expanded gene families in *T. larimichthysi* revealed over-representation in pathways primarily associated with host-pathogen interactions, immune evasion, metabolic regulation, and cellular signaling (Fig. 4C). These enrichments suggest that gene family expansions in *T. larimichthysi* are linked to enhanced mechanisms for host invasion, immune modulation, and environmental adaptation, potentially facilitating its parasitism in marine fish hosts.

### Ethics statement

All fish sampling and parasite collection procedures were conducted in accordance with institutional and national guidelines for animal care and use. The experimental protocol was reviewed and approved by the Animal Ethics Committee of Fujian Academy of Agricultural Sciences, Agriculture Quality Standards and Testing Technology Research Institute (approval number: ZBLISC2025-01).

## Data Records

The raw HiFi, Hi-C reads of *T. larimichthysi* and RNA-seq reads used for gene structure annotation have been deposited in the NCBI Sequence Read Archive (SRA) under accession numbers of SRR36309045, SRR36309044 and SRR38925527, respectively^67^. The genome assemblies have been deposited at GeneBank with accession numbers of GCA_054345675.1^68^. Additionally, we have deposited the genome assemblies and gene annotation results in Figshare (https://doi.org/10.6084/m9.figshare.30898094)^69^.

## Technical Validation

The *T. larimichthysi* genome assembly demonstrates reference-grade quality through rigorous validation metrics, underpinned by highly reliable PacBio HiFi and Hi-C sequencing data. The 4.96 Gb of HiFi reads exhibited exceptional quality, with a mean read length of 19,778 bp and N50 of 20,410 b, ensuring long, accurate reads ideal for resolving complex parasitic genome. The final assembly is completely gap-free (no ambiguous N bases) and resolved at the chromosome level, comprising exactly 35 pseudochromosomes with 97.96% of the sequence anchored via Hi-C scaffolding. Telomeric completeness is outstanding: canonical TTAGGG/CCCTAA repeats were detected at 58 chromosomal ends, achieving telomere-to-telomere (T2T) resolution for 25 of 35 chromosomes. Realignment of the original HiFi reads to the assembly yielded a near-perfect mapping rate of 99.99%, confirming exceptional base-level accuracy and structural continuity. BUSCO analysis using the Euglenozoa_odb10 dataset exceeded 99% completeness in both genome and protein modes, underscoring near-complete capture of conserved eukaryotic genes. These features, reliable high-accuracy HiFi data, gap-free status, chromosome-scale resolution, extensive telomeric capture, perfect read concordance, and superior BUSCO scores, collectively establish this as one of the highest-quality trypanosomatid genomes reported to date.

## Data Availability

The bioinformatic tools, including version and parameters, that were used in this study have been described in the Method section. No customized script was used.

## References

[1] Stevens, J., Noyes, H., Schofield, C. & Gibson, W. The molecular evolution of Trypanosomatidae. (2001). 10.1016/s0065-308x(01)48003-1 11013754

[2] Vickerman, K. J. I. J. f. P. The evolutionary expansion of the trypanosomatid flagellates. **24**, 1317-1331 (1994). 10.1016/0020-7519(94)90198-8 7729984

[3] Desquesnes, M. *et al*. A review on the diagnosis of animal trypanosomoses. **15**, 64 (2022). 10.1186/s13071-022-05190-1 PMC8858479 35183235

[4] Thompson, C. K., Godfrey, S. S., Thompson, R. A. J. I. J. f. P. P. & Wildlife. Trypanosomes of Australian mammals: a review. **3**, 57-66 (2014). 10.1016/j.ijppaw.2014.02.002 PMC4142263 25161902

[5] Aregawi, W. G., Agga, G. E., Abdi, R. D., Büscher, P. J. P. & vectors. Systematic review and meta-analysis on the global distribution, host range, and prevalence of Trypanosoma evansi. **12**, 67 (2019). 10.1186/s13071-019-3311-4 PMC6357473 30704516

[6] Hoare, C. A. The trypanosomes of mammals. A zoological monograph. (1972).

[7] Büscher, P., Cecchi, G., Jamonneau, V. & Priotto, G. J. T. L. Human african trypanosomiasis. **390**, 2397-2409 (2017). 10.1016/S0140-6736(17)31510-6 28673422

[8] Rassi, A. & Marin-Neto, J. A. J. T. L. Chagas disease. **375**, 1388-1402 (2010). 10.1016/S0140-6736(10)60061-X 20399979

[9] Desquesnes, M. *et al*. Trypanosoma evansi and surra: a review and perspectives on transmission, epidemiology and control, impact, and zoonotic aspects. **2013**, 321237 (2013). 10.1155/2013/321237 PMC3789323 24151595

[10] Horn, D. J. M. & parasitology, b. Antigenic variation in African trypanosomes. **195**, 123-129 (2014). 10.1016/j.molbiopara.2014.05.001 PMC4155160 24859277

[11] Taylor, J. E. & Rudenko, G. J. T. I. G. Switching trypanosome coats: what’s in the wardrobe? **22**, 614-620 (2006). 10.1016/j.tig.2006.08.003 16908087

[12] Khan, R. J. C. J. O. Z. Susceptibility of marine fish to trypanosomes. **55**, 1235-1241 (1977). 10.1139/z77-162 902173

[13] Khan, R., Barrett, M. & Murphy, J. J. C. J. O. Z. Blood parasites of fish from the northwestern Atlantic Ocean. **58**, 770-781 (1980).

[14] Grybchuk-Ieremenko, A., Losev, A., Kostygov, A. Y., Lukes, J. & Yurchenko, V. J. F. P. High prevalence of trypanosome co-infections in freshwater fishes. **61**, 495 (2014). 25651690

[15] Lom, J. Biology of the trypanosomes and trypanoplasms of fish. (1979).

[16] Chong, R. S.-M. in *Aquaculture pathophysiology* 601-609 (Elsevier, 2022).

[17] Losev, A., Grybchuk-Ieremenko, A., Kostygov, A. Y., Lukeš, J. & Yurchenko, V. J. P. r. Host specificity, pathogenicity, and mixed infections of trypanoplasms from freshwater fishes. **114**, 1071-1078 (2015). 10.1007/s00436-014-4277-y 25544706

[18] McAllister, M. Pathophysiology in fish trypanosomiasis: The interaction between Trypanosoma carassii and goldfish erythropoietic system during the course of infection. (2019).

[19] Khan, R. J. C. J. o. Z. Pathogenesis of Trypanosoma murmanensis in marine fish of the northwestern Atlantic following experimental transmission. **63**, 2141-2144 (1985).

[20] Hemmingsen, W. & MacKenzie, K. The parasite fauna of the Atlantic cod, Gadus morhua L. (2001).

[21] Lemos, M. *et al*. Phylogenetic and morphological characterization of trypanosomes from Brazilian armoured catfishes and leeches reveal high species diversity, mixed infections and a new fish trypanosome species. **8**, 573 (2015). 10.1186/s13071-015-1193-7 PMC4636803 26546294

[22] Gu, Z. *et al*. Morphological and genetic differences of Trypanosoma in some Chinese freshwater fishes: difficulties of species identification. **101**, 723-730 (2007). 10.1007/s00436-007-0536-5 17558522

[23] Le Roux, C., Cook, C. A., Netherlands, E. C., Truter, M. & Smit, N. J. J. P. Molecular and morphological characterization of one known and three new species of fish parasitic Trypanosoma Gruby, 1972 from the south coast of South Africa. 1-20 (2025). 10.1017/S0031182025000496 PMC12278017 40194954

[24] Yang, X. *et al*. Identification of a new fish trypanosome from the large yellow croaker (Larimichthys crocea) and description of its impact on host pathology, blood biochemical parameters and immune responses. **32**, 1 (2025). 10.1051/parasite/2024078 PMC11752739 39840893

[25] Yang, Z., Lu, N. & Zhai, L. J. A. I. Study on production strategies for marine aquaculture in China at different scales: a case study of large yellow croaker (Larimichthys crocea). **33**, 172 (2025).

[26] Ye, G. *et al*. Could the wild population of Large Yellow Croaker Larimichthys crocea (Richardson) in China be restored? A case study in Guanjingyang, Fujian. *China* **33**, 24 (2020).

[27] Yan, L.-t. *et al*. Reproductive dynamics of the large yellow croaker Larimichthys crocea (Sciaenidae), a commercially important fishery species in China. **9**, 868580 (2022).

[28] Yao, J. X. *et al*. Stability of population genetic structure in large yellow croaker (Larimichthys crocea): Insights from temporal, geographical factors, and artificial restocking processes. **14**, e70207 (2024). 10.1002/ece3.70207 PMC11347937 39193171

[29] Jiang, Z. *et al*. Genomic Approaches to Enhance Disease Resistance in Large Yellow Croaker: Recent Progress and Future Perspectives. **17**, e70084 (2025).

[30] Chen, S., Su, Y., Hong, W. J. A. i. C. s. s. & trends, m. Aquaculture of the large yellow croaker. 297-308 (2018).

[31] Xu, S., Liu, Q., Yang, T., Han, Z. J. J. o. G. & Genomics. A telomere-to-telomere genome assembly of the large yellow croaker provides insights into evolution of golden-yellow coloration (2025). 10.1016/j.jgg.2025.09.006 40975488

[32] Wang, J.-F. *et al*. Characterization of a trypanosome from large yellow croaker (Larimichthys crocea), cage-cultured in seawater, in China. *Aquaculture Reports* **43** 10.1016/j.aqrep.2025.102868 (2025).

[33] Cheng, H., Concepcion, G. T., Feng, X., Zhang, H. & Li, H. Haplotype-resolved de novo assembly using phased assembly graphs with hifiasm. *Nature methods* **18**, 170–175, 10.1038/s41592-020-01056-5 (2021). PMC7961889 33526886

[34] Xu, M. *et al*. TGS-GapCloser: a fast and accurate gap closer for large genomes with low coverage of error-prone long reads. **9**, giaa094 (2020). 10.1093/gigascience/giaa094 PMC7476103 32893860

[35] Servant, N. *et al*. HiC-Pro: an optimized and flexible pipeline for Hi-C data processing. *Genome biology* **16**, 259, 10.1186/s13059-015-0831-x (2015). PMC4665391 26619908

[36] Langmead, B. & Salzberg, S. L. Fast gapped-read alignment with Bowtie 2. *Nature methods* **9**, 357–359, 10.1038/nmeth.1923 (2012). PMC3322381 22388286

[37] Durand, N. C. *et al*. Juicebox Provides a Visualization System for Hi-C Contact Maps with Unlimited Zoom. *Cell Syst* **3**, 99–101, 10.1016/j.cels.2015.07.012 (2016). PMC5596920 27467250

[38] Krzywinski, M. *et al*. Circos: an information aesthetic for comparative genomics. *Genome research* **19**, 1639–1645, 10.1101/gr.092759.109 (2009). PMC2752132 19541911

[39] Benson, G. Tandem repeats finder: a program to analyze DNA sequences. *Nucleic acids research* **27**, 573–580, 10.1093/nar/27.2.573 (1999). PMC148217 9862982

[40] Simao, F. A., Waterhouse, R. M., Ioannidis, P., Kriventseva, E. V. & Zdobnov, E. M. BUSCO: assessing genome assembly and annotation completeness with single-copy orthologs. *Bioinformatics* **31**, 3210–3212, 10.1093/bioinformatics/btv351 (2015). 26059717

[41] Li, H. Minimap2: pairwise alignment for nucleotide sequences. *Bioinformatics* **34**, 3094–3100, 10.1093/bioinformatics/bty191 (2018). PMC6137996 29750242

[42] Li, H. *et al*. The Sequence Alignment/Map format and SAMtools. *Bioinformatics* **25**, 2078–2079, 10.1093/bioinformatics/btp352 (2009). PMC2723002 19505943

[43] Stanke, M. *et al*. AUGUSTUS: ab initio prediction of alternative transcripts. *Nucleic Acids Research* **34**, W435–439, 10.1093/nar/gkl200 (2006). PMC1538822 16845043

[44] Slater, G. S. & Birney, E. Automated generation of heuristics for biological sequence comparison. *BMC bioinformatics* **6**, 31, 10.1186/1471-2105-6-31 (2005). PMC553969 15713233

[45] Birney, E., Clamp, M. & Durbin, R. GeneWise and Genomewise. *Genome Research* **14**, 988–995, 10.1101/gr.1865504 (2004). PMC479130 15123596

[46] Haas, B. J. *et al*. Automated eukaryotic gene structure annotation using EVidenceModeler and the Program to Assemble Spliced Alignments. *Genome Biology* **9**, R7, 10.1186/gb-2008-9-1-r7 (2008). PMC2395244 18190707

[47] Kim, D., Paggi, J. M., Park, C., Bennett, C. & Salzberg, S. L. Graph-based genome alignment and genotyping with HISAT2 and HISAT-genotype. *Nature biotechnology* **37**, 907–915, 10.1038/s41587-019-0201-4 (2019). PMC7605509 31375807

[48] Kovaka, S. *et al*. Transcriptome assembly from long-read RNA-seq alignments with StringTie2. *Genome biology* **20**, 278, 10.1186/s13059-019-1910-1 (2019). PMC6912988 31842956

[49] Johnson, L. S., Eddy, S. R. & Portugaly, E. Hidden Markov model speed heuristic and iterative HMM search procedure. *BMC bioinformatics* **11**, 431, 10.1186/1471-2105-11-431 (2010). PMC2931519 20718988

[50] Jones, P. *et al*. InterProScan 5: genome-scale protein function classification. *Bioinformatics* **30**, 1236–1240, 10.1093/bioinformatics/btu031 (2014). PMC3998142 24451626

[51] Punta, M. *et al*. The Pfam protein families database. *Nucleic acids research* **40**, D290–301, 10.1093/nar/gkr1065 (2012). PMC3245129 22127870

[52] Ashburner, M. *et al*. Gene ontology: tool for the unification of biology. The Gene Ontology Consortium. *Nature genetics* **25**, 25–29, 10.1038/75556 (2000). PMC3037419 10802651

[53] Cantalapiedra, C. P., Hernandez-Plaza, A., Letunic, I., Bork, P. & Huerta-Cepas, J. eggNOG-mapper v2: Functional Annotation, Orthology Assignments, and Domain Prediction at the Metagenomic Scale. *Molecular biology and evolution* **38**, 5825–5829, 10.1093/molbev/msab293 (2021). PMC8662613 34597405

[54] Ogata, H. *et al*. KEGG: Kyoto Encyclopedia of Genes and Genomes. *Nucleic acids research* **27**, 29–34, 10.1093/nar/27.1.29 (1999). PMC148090 9847135

[55] Galperin, M. Y. *et al*. COG database update: focus on microbial diversity, model organisms, and widespread pathogens. *Nucleic acids research* **49**, D274–D281, 10.1093/nar/gkaa1018 (2021). PMC7778934 33167031

[56] Rodriguez-Tome, P., Stoehr, P. J., Cameron, G. N. & Flores, T. P. The European Bioinformatics Institute (EBI) databases. *Nucleic acids research* **24**, 6–12 (1996). 10.1093/nar/24.1.6 PMC145572 8594602

[57] Flynn, J. M. *et al*. RepeatModeler2 for automated genomic discovery of transposable element families. *Proceedings of the National Academy of Sciences of the United States of America* **117**, 9451–9457, 10.1073/pnas.1921046117 (2020). PMC7196820 32300014

[58] Chen, N. Using RepeatMasker to identify repetitive elements in genomic sequences. *Current protocols in bioinformatics* **Chapter 4**, Unit 4 10, 10.1002/0471250953.bi0410s05 (2004). 18428725

[59] Emms, D. M. & Kelly, S. OrthoFinder: phylogenetic orthology inference for comparative genomics. *Genome biology* **20**, 238, 10.1186/s13059-019-1832-y (2019). PMC6857279 31727128

[60] Katoh, K. & Standley, D. M. MAFFT multiple sequence alignment software version 7: improvements in performance and usability. *Molecular biology and evolution* **30**, 772–780, 10.1093/molbev/mst010 (2013). PMC3603318 23329690

[61] Stamatakis, A. RAxML version 8: a tool for phylogenetic analysis and post-analysis of large phylogenies. *Bioinformatics* **30**, 1312–1313, 10.1093/bioinformatics/btu033 (2014). PMC3998144 24451623

[62] Letunic, I. & Bork, P. Interactive Tree Of Life (iTOL) v5: an online tool for phylogenetic tree display and annotation. *Nucleic acids research* **49**, W293–W296, 10.1093/nar/gkab301 (2021). PMC8265157 33885785

[63] Yang, Z. PAML 4: phylogenetic analysis by maximum likelihood. *Molecular biology and evolution* **24**, 1586–1591, 10.1093/molbev/msm088 (2007). 17483113

[64] Kumar, S. *et al*. TimeTree 5: An Expanded Resource for Species Divergence Times. *Molecular biology and evolution* **39**, 10.1093/molbev/msac174 (2022). PMC9400175 35932227

[65] De Bie, T., Cristianini, N., Demuth, J. P. & Hahn, M. W. CAFE: a computational tool for the study of gene family evolution. *Bioinformatics* **22**, 1269–1271, 10.1093/bioinformatics/btl097 (2006). 16543274

[66] Wickham, H. *ggplot2: Elegant Graphics for Data Analysis*. (Springer-Verlag New York, 2016).

[67] *NCBI Sequence Read Archive* https://identifiers.org/ncbi/insdc.sra:SRP651431 (2026).

[68] *NCBI GenBank* https://identifiers.org/ncbi/insdc.gca:GCA_054345675.1 (2026).

[69] Lin, L. Genome assembly and gene annotation of *Trypanosoma larimichthysi*. *figshare*10.6084/m9.figshare.30898094 (2026).

